# Machine learning based personalized promotion strategy of piglets weaned per sow per year in large-scale pig farms

**DOI:** 10.1186/s40813-022-00280-z

**Published:** 2022-08-10

**Authors:** Xingdong Zhou, Ran Guan, Hongbo Cai, Pei Wang, Yongchun Yang, Xiaodu Wang, Xiaowen Li, Houhui Song

**Affiliations:** 1 Key Laboratory of Applied Technology On Green-Eco-Healthy Animal Husbandry of Zhejiang Province, Zhejiang Provincial Engineering Laboratory for Animal Health Inspection and Internet Technology, Zhejiang International Science and Technology Cooperation Base for Veterinary Medicine and Health Management, China-Australia Joint Laboratory for Animal Health Big Data Analytics, College of Animal Science and Technology and College of Veterinary Medicine of Zhejiang Agriculture and Forestry University, 666 Wusu Street, Lin’an District, Hangzhou, 311300 Zhejiang People’s Republic of China; 2 Shandong New Hope Liuhe Agriculture and Animal Husbandry Technology Co., Ltd (NHLH Academy of Swine Research), No. 6596 Dongfanghong East Road Yuanqiao Town, Dezhou, 253000 Shandong People’s Republic of China; 3 Intelligent Engine Department, The Ant Financial (Hang Zhou, Network Technology Co., Ltd, A Space, No. 569 Xixi Road, Xihu District, Hangzhou, 310023 Zhejiang People’s Republic of China; 4 Beijing Center for Animal Disease Control and Prevention, No. 19 Xiangrui Street, Biological Medicine Base, Daxing District, Beijing, 102629 People’s Republic of China

**Keywords:** PSY, Machine learning, Correlation coefficient, Gradient boosting regressor model, Personalized improvement

## Abstract

**Background:**

The purpose of this study was to analyze the relationship between different productive factors and piglets weaned per sow per year (PSY) in 291 large-scale pig farms and analyze the impact of the changes in different factors on PSY. We chose nine different algorithm models based on machine learning to calculate the influence of each variable on every farm according to its current situation, leading to personalize the improvement of the impact in the specific circumstances of each farm, proposing a production guidance plan of PSY improvement for every farm. According to the comparison of mean absolute error (MAE), 95% confidence interval (CI) and R^2^, the optimal solution was conducted to calculate the influence of 17 production factors of each pig farm on PSY improvement, finding out the bottleneck corresponding to each pig farm. The level of PSY was further analyzed when the bottleneck factor of each pig farm changed by 0.5 standard deviation (SD).

**Results:**

17 production factors were non-linearly related to PSY. The top five production factors with the highest correlation with PSY were the number of weaned piglets per litter (WPL) (0.6694), mating rate within 7 days after weaning (MR7DW) (0.6606), number of piglets born alive per litter (PBAL) (0.6517), the total number of piglets per litter (TPL) (0.5706) and non-productive days (NPD) (− 0.5308). Among nine algorithm models, the gradient boosting regressor model had the highest R^2^, smallest MAE and 95% CI, applied for personalized analysis. When one of 17 production factors of 291 large-scale pig farms changed by 0.5 SD, 101 pig farms (34.7%) can increase 1.41 PSY (compared to its original value) on average by adding the production days, and 60 pig farms (20.6%) can increase 1.14 PSY on average by improving WPL, 45 pig farms (15.5%) can increase 1.63 PSY by lifting MR7DW.

**Conclusions:**

The main productive factors related to PSY included WPL, MR7DW, PBAL, TPL and NPD. The gradient boosting regressor model was the optimal method to individually analyze productive factors that are non-linearly related to PSY.

**Supplementary Information:**

The online version contains supplementary material available at 10.1186/s40813-022-00280-z.

## Background

Piglets weaned per sow per year (PSY) is a key factor to evaluate the productivity performance of pig farms, which has been widely used in the pig industry for more than 30 years [1]. By using information management systems in large-scale pig farms (such as the Huiyangzhu system used by Shandong New Hope Liuhe Group Co., Ltd.), managers can obtain PSY and relevant production factors in time to make better management decisions and goals [2]. To improve PSY, a series of measures need to be taken to improve reproductive performance of sows, including emphasizing the management of replacement gilts to improve their lifetime production efficiency, balancing feed nutrition during lactation to increase weaning weight, strengthening piglet care within three days of farrowing to reduce pre-weaning mortality and paying attention to personnel skill training to implement strategies correctly [3–5].

The genetic progress of the pig industry has significantly improved PSY and related performance [6]. Some countries have PSY approaching or even exceeding 30 [7], resulting in a great promotion of the global protein supply. However, there is a lot of room for improvement in this regard in China. In production practice, if you can choose the factor that has the greatest impact on PSY among the related factors of PSY, improving this factor will achieve a multiplier effect with half the effort [8]. Algorithm models (including linear and nonlinear models) may provide an effective technical method to solve this problem. In the field of pig breeding, the algorithm model has been gradually applied to pig feed formula optimization, breeding analysis, disease transmission dynamics and major infectious disease prediction [9–12]. In terms of management, it can also help managers to analyze market conditions, evaluate sales opportunities and formulate sales plans [13, 14]. Linear models have been often used in the analysis of production data, including PSY. For example, Munsterhjelm et al. [15] used multiple linear regression to study the relationship between farm welfare and sow reproductive performance. Sanglard et al. [16] estimated the heritability and genetic correlation between the sample-to-positive (S/P) ratio and reproductive performance of breeding pigs after vaccination with porcine reproductive and respiratory syndrome (PRRS) vaccine with the BayesCo linear regression model. BayesB linear regression model was used to analyze the bivariate genome-wide association between PRRS antibody response and reproductive traits [17]. However, when the data performance is not completely linear, the use of a linear model has limitations, resulting in poor effect and affecting the accuracy of prediction. Although the contribution of each independent variable can be estimated from both linear and nonlinear models, the target factor of each farm can be accurately calculated by nonlinear models instead of the overall trend estimation by linear models. Therefore, nonlinear models have advantages in processing production data.

The purpose of this study was to analyze the key production factors of 291 large-scale pig farms and their relationship with PSY through machine learning, analyze the correlation between factors, and find out the production factors with high correlation with PSY. Through the optimal algorithm model obtained by the cross-continuation of machine learning, the expected growth value of PSY in each farm is calculated when all production indicators increase by 0.5 standard deviations (SD), and a personalized PSY promotion strategy is given. To our knowledge, no previous study has evaluated the PSY using gradient boosting regressor model.

## Methods

### Farm description

The study did not require approval from the Ethics Committee on Animal Use because no animal was handled. This study involved 648,826 breeding sows of 291 pig breeding farms from 79 large-scale breeding subsidiaries. They fulfilled the following inclusion criteria, which were (1) having a population of 750 or more sows, (2) using the internal data management system of the company, and completing data records.

The automatic feeding system, mechanical ventilation system, formula of standardized feed and mating schedule of these farms were described in a previous study [8]. The farms were from 20 provinces, located in seven regions of the country, namely, East China (40.5%), North China (3.8%), South China (15.2%), Central China (8.9%), Northwest (15.2%), Northeast (5.1%) and Southwest (11.4%) regions.

### Data collection and manipulation

The collection and authorization of these production data in this study were described before [8]. This study analyzed 291 large-scale (750–5,800 sows) pig farms for the whole year of 2021. All variables were recorded at the herd level, taking into account previous studies on pig farms as they demonstrate herd performance and are important to farm economics. All 17 production factors in the original data system were used to analyze the relationship with PSY and subsequent analysis.

### Definitions

Grandparent (GP) farm means the pig farm where only purebred sows are kept, which were from a Landrace pure line. Parent stock (PS) farm means the pig farm where the crossbred sows (F1 offspring) are kept, which were from a cross between the Landrace pure line and a Large White pure line. All crossbred sows were produced from Large White female and a Landrace male.

Due to producer confidentiality, the absolute value of PSY was normalized from 0 to 1 (100%). For non-digital variable, farm type follows the formal definition below:

$$farm\,type\left\{\begin{array}{c}1\,\,\,\, if \,\,type=PS\\ 0 \,\,\,\,if \,\,type=GP\end{array}\right.$$

### Statistics analysis

All analyses were conducted with the python programming language in PyCharm 2021.3.2 (Community Edition). The farm was considered an experimental unit. In order to reduce the noise in raw data, abnormal data points were deleted. Each record was the production data of the farm for the whole year of 2021. Due to the impact of production operations or epidemics, the farrowing rate (FR) of some farms was 0, indicating that the farm has not delivered piglets in 2021; the number of piglets born alive per litter (PBAL) was 0, indicating that the farm cannot provide PBAL and cannot calculate the PSY. Therefore, farm with 0 FR and 0 PBAL are excluded as abnormal data. Spearman's rank correlation analysis between 18 variables (including PSY) was performed to construct the Correlation Coefficient Matrix. The correlation between each variable and PSY and the collinearity between each variable were analyzed through the correlation coefficient matrix.

Nine machine learning models were applied to learn and predict the PSY of the pig farm with the sklearn algorithm module, which were Gradient Boosting Regressor, HistGradient Boosting Regressor, Extra Trees Regressor, Random Forest Regressor, Bayesian Ridge, Linear Regression, Bagging Regressor, AdaBoost Regressor, ElasticNet. Leave-One-Out Cross-validation (LOO-CV) was applied for machine learning and prediction. In each cycle, 290 farms were taken for training, and one farm was used for prediction. A total of 291 models were established in 291 cycles. Finally, the prediction results were aggregated to complete the final effect evaluation.

Also, machine learning model predictions were performed to individually find the PSY improvement bottleneck of each farm. Specifically, for each pig farm, we modified every factor separately, which was increased or decreased by 0.5 standard deviation of the factor. Then, re-predicted the updated data to compare the modification plan that maximized the improvement of PSY. The formal definition for prediction was as follows:

$$argmax \,\,pred\left({X}_{i}^{^{\prime}}\right)$$

$$s.t. {x}_{ij}^{^{\prime}}={x}_{ij}+\delta \cdot t\cdot {std}_{j}\cdot {w}_{ij}$$

$${w}_{ij}\in \left\{0, 1\right\},\forall j\in \{1, \dots , M\},\forall i\in \{1, \dots , N\}$$

$${\sum }_{j=1}^{M}{w}_{ij}=1,\forall j\in \{1, \dots , M\} ,\forall i\in \{1, \dots , N\}$$

$$t\in \left\{-1, 1\right\}$$

$${X}_{i}=\left[{x}_{i1},\cdots ,{x}_{ij},\cdots ,{x}_{iM}\right]\in {\mathbb{R}}^{M},\forall i\in \left\{1, \dots , N\right\},\forall j\in \{1, \dots , M\}$$

$${X}_{i}^{^{\prime}}=\left[{x}_{i1}^{^{\prime}},\cdots ,{x}_{ij}^{^{\prime}},\cdots ,{x}_{iM}^{^{\prime}}\right]\in {\mathbb{R}}^{M},\forall i\in \{1, \dots , N\},\forall j\in \{1, \dots , M\}$$

where *N* presents the total number of pig farms; *M* presents the total number of variables. *X*_*ij*_ means the value of variable number *j* in the number *i* farm; *X*_*i*_ means a vector consisting of all variables of the number *i* farm; *x*^*’*^_*ij*_ means the result of changing *x*_*ij*_ by a small amount. *std*_*j*_ is the standard deviation of the number* j* variable; *t* is used to control the direction of change (increase or decrease);* δ* is the change amplitude parameter.

Residual analysis was performed in Prism 8 (8.4.0) for normality test and in Microsoft Excel (16.61.1) for homoscedastic test.

## Results

**Fig. 1:**
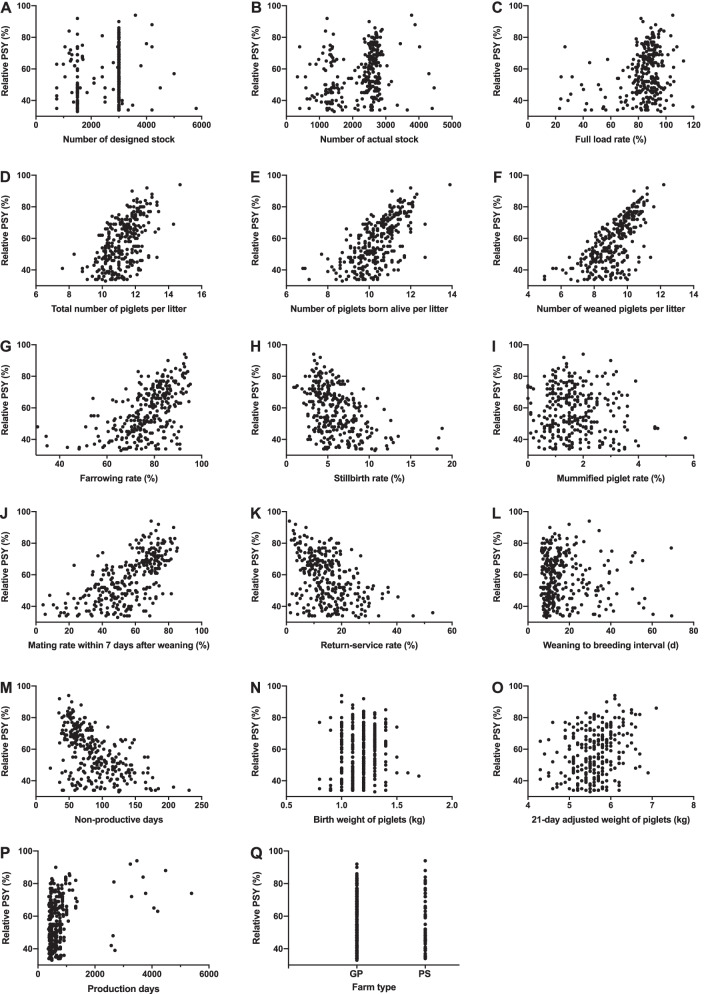
Relationship between 17 production factors and PSY in 291 large-scale pig farms

Figure 1 showed that 17 production factors were non-linearly distributed with PSY, in which total number of piglets per litter (TPL), PBAL, number of weaned piglets per litter (WPL), FR and mating rate within 7 days after weaning (MR7DW) were positively correlated with PSY. Instead, the stillbirth rate, return-service rate, weaning to breeding interval and NPD were negatively correlated with PSY. And the correlation of the other six were not clear. Many of these production factors limited the upper bound of PSY (Fig. 1DEFGHJKLM), indicating that they could be the bottleneck of PSY improvement on this farm.

**Fig. 2:**
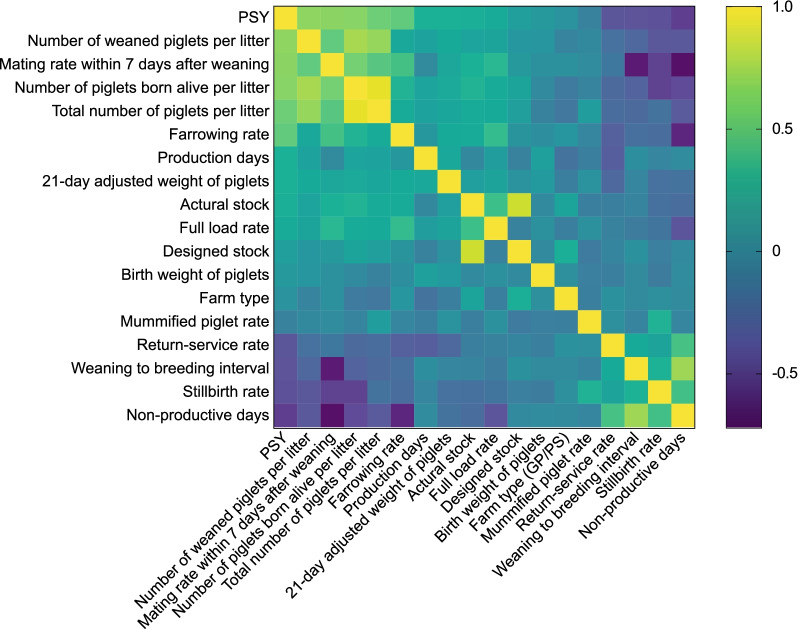
Correlation coefficient matrix of 18 production factors in 291 large-scale pig farms

The correlation coefficient matrix of 18 parameters in 291 large-scale pig farms is shown in Fig. 2. The production factors with a strong correlation (≥ 0.8000) between two pairs included TPL versus PBAL (0.9440), followed by design stock versus actual stock (0.8516). The top five production factors with the highest correlation with PSY were WPL (0.6694), MR7DW (0.6606), PBAL (0.6517), TPL (0.5706) and NPD (− 0.5308).

Comparing the mean absolute error (MAE), 95% confidence interval (CI), R^2^, mean square error (MSE) and mean absolute percentage error (MAPE) of nine algorithm models, the gradient boosting regressor model had the smallest MAE, the narrowest 95% CI and the highest R^2^; meanwhile, the MSE and MAPE were relatively low (Table 1). The residuals of gradient boosting regressor are normally distributed (*P* value > 0.05 by D’Agootino-Pearson test) and homoscedastic (*P* value > 0.05 by Breusch-Pagan test).

**Table 1:** Evaluation factors and 95% confidence interval of different models

Model	MAE	95% CI	R^2^	MSE	MAPE
Gradient boosting regressor	1.6047	4.262	0.7432	5.0377	10.70%
Hist gradient boosting regressor	1.6211	4.5973	0.7399	4.8847	10.68%
Extra trees regressor	1.6318	4.488	0.7353	5.0112	10.84%
Random forest regressor	1.7397	4.642	0.7103	5.5809	11.64%
Bayesian ridge	1.7691	4.6014	0.7098	5.5517	11.73%
Linear regression	1.7744	4.8353	0.7048	5.6470	11.76%
Bagging regressor	1.8329	5.3001	0.7034	6.0023	12.06%
Ada Boost regressor	2.0326	5.08	0.6679	6.4055	12.84%
Elastic net	2.36	6.0404	0.5354	8.8888	15.38%

MAE* =* mean absolute error; CI = confidence interval; MSE = mean square error; MAPE = mean absolute percentage error

**Fig. 3:**
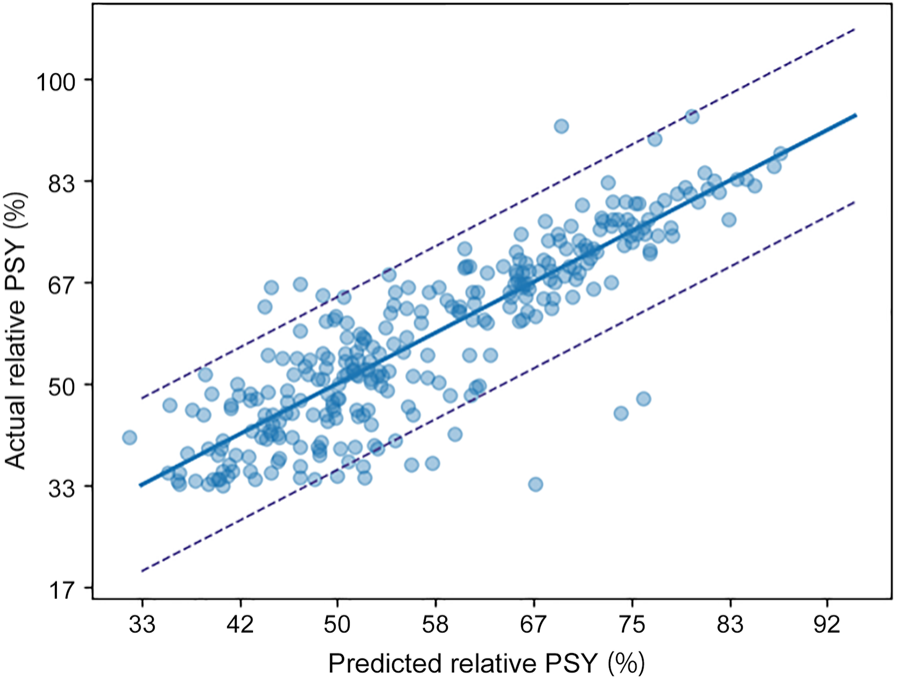
Scatter plot of actual versus predicted values of PSY derived from gradient boosting regressor model. The solid line represents the regression curve, and dotted lines represent the 95% confidence interval

Figure 3 showed that the predicted PSY calculated by the gradient boosting regressor model was highly confident with the actual PSY. 95.19% (277/291 pig farms) of the predicted PSY values fell within the 95% CI. The MAE value was 1.6047, which indicated that the average difference between the results predicted using the model and the actual PSY was 1.6047.

The changes of 17 production factors of 291 large-scale pig farms at 0.5 SD absolute value were calculated by the gradient boosting regressor model, respectively. And the bottleneck factor and corresponding PSY promotion value of each pig farms were obtained (see Additional file 1). In addition to the return-service rate and farm type, a 0.5 SD change of 15 other production factors can increase PSY to various levels (0.15 PSY-3.31 PSY) (Table 2). In one third of the pig farms, an absolute increase of 0.5 SD in the number of production days can increase 0.29–2.92 PSY. With an increase of WPL, one fifth of the pig farms can improve an average of 1.14 PSY. One sixth of the pig farms can improve an average of 1.63 PSY with the increase of MR7DW. These three factors accounted for 70.8% of all pig farms. Among all factors, weaning to breeding interval had the largest average improvement (1.84 PSY).

**Table 2:** Personalized bottleneck calculated by gradient boosting regressor model*

Factors	Number of pig farms	The proportion of pig farms	0.5 SD	Average	Median	Max	Min
Production days	101	34.7%	163.60	1.41	1.44	2.92	0.29
Number of weaned piglets per litter	60	20.6%	0.46	1.14	1.15	1.97	0.22
Mating rate within 7 days after weaning	45	15.5%	6.99%	1.63	1.58	3.31	0.50
Farrowing rate	24	8.2%	4.06%	1.01	1.07	1.76	0.28
Designed stock	17	5.8%	299.90	1.33	1.29	2.37	0.61
Number of piglets born alive per litter	15	5.2%	0.40	1.04	1.05	1.45	0.61
Weaning to breeding interval	11	3.8%	4.39	1.84	1.82	2.57	1.16
Non-productive days	4	1.4%	14.15	1.35	1.06	2.93	0.35
Actual stock	3	1.0%	300.01	1.56	1.50	2.74	0.42
Full load rate	3	1.0%	4.58%	0.32	0.32	0.36	0.28
Birth weight of piglets	2	0.7%	0.06	0.67	0.67	1.01	0.33
Mummified piglet rate	2	0.7%	0.36%	0.27	0.27	0.38	0.15
Total number of piglets per litter	2	0.7%	0.40	0.80	0.80	1.21	0.38
21-day adjusted weight of piglets	1	0.3%	0.18	0.34	0.34	0.34	0.34
Stillbirth rate	1	0.3%	1.01%	0.28	0.28	0.28	0.28

For PSY improvement of 291 large-scale pig farms, each factor was increased by the absolute value of 0.5 standard deviation

SD* =* standard deviation

*Return-service rate and farm type did not affect the PSY improvement by gradient boosting regressor

## Discussion

The production data of this study came from 291 large-scale pig farms in 79 subsidiaries of an agri-food group. Although the design of pig farms, feed quality and standard operating procedure (SOP) was consistent, there were differences in scale, breeds, hygiene and personnel implementation, which may affect the performance of piglets and sows [18–20]. In order to reduce the influence of external factors as much as possible on the basis of ensuring the amount of data, the following measures have been taken: (1) we selected large-scale farms for this study with more than 750 breeding sows. Koketsu and Lida [5] showed that large-scale pig farms had more advanced facilities, more human resources and a higher level of genetic improvement than small ones. (2) The statistical factors were expressed as the average value of all sows per pig farm within one year (2021), avoiding the interference of seasonal effects [20]. To avoid respondents' bias, this study used only objective production data [21]. Although there may be some errors in data input and there is no distinction of parity (69.4% of pig farms were put into operation in 2020 and later, mainly because the parity is generally low), the data trend was still useful of reference, and our conclusions were in line with the calculation formula of PSY.

With the recording and storage of production "big data", the scale, standardisation and modernization of pig farms will be the future development direction of the breeding industry. In production management software, only preliminary descriptive analysis was generally carried out, and most producers only used these data to generate basic production performance and working lists [22]. Deep mining the meaning of data and using data analysis-based pig herd management can help producers and veterinarians in large-scale pig farms maximize the potential productivity of pigs and improve economic benefits [5].

Because the cycle of pig breeding was relatively long, the production chain was complex, and it was easy to be affected by factors such as environment, nutrition and disease. Greater computing power and more complex mathematical methods were required to meet the needs of epidemiological investigation, disease prediction, production data analysis, etc. Using a Rimpuf simulation model to simulate airborne transmission of foot-and-mouth disease, Sørensen et al. [23] found that the number of pigs infected with foot-and-mouth disease and the source concentration of airborne viruses in France in 1981 were likely much higher than historical record. The outbreak of foot-and-mouth disease in Denmark in 1982 may have been due to the underreporting of the swine epidemic in the former Democratic Germany Republic (GDR). La et al. [24] combined hydrodynamic simulations with β-Combined Poisson model to successfully simulated the aerosol transmission of PRRSV, and the predicted infection probability matched the observed infection well. Evans et al. [9] found that the reintroduction, persistence and extinction of PRRSV played a key role in the intra-transmission of PRRSV through the random mathematical modeling of piglet herds.

The nine algorithm models used in this study had different characteristics. Linear Regression is a simple and widely used model for overall trends calculation and prediction but cannot calculate individualized results for each farm. All the other eight models are nonlinear. Compared with linear regression, they can better learn the nonlinear relationship between factors and PSY or the combined effect of each factor. Bayesian ridge regression model combines the advantages of Bayesian linear regression and ridge regression models, which is that regularization parameters can be learned automatically, avoiding overfitting or underfitting. It can be applied not only to the fitting of normal data but also to the fitting of morbid or abnormal data. It can also solve the problem of overfitting in maximum likelihood estimation, and the utilization rate of logarithmic data samples was 100%; only using training samples can effectively and accurately determine the complexity of the model and can be used to predict the litter size [25]. Bagging Regressor can solve the overfitting problem through ensemble, suitable for small data sets. Elastic Net optimizes overfitting and underfitting problems by combining L1 + L2 (linear combination between Ridge and Lasso) regularization methods.

Tree models (including Random Forest Regressor, Ada Boost Regressor, Extra Trees Regressor, Gradient Boosting Regressor, Hist Gradient Boosting Regressor) can learn the combination of key factors that affect PSY and calculate the PSY of the farm according to the PSY fitting of other similar samples. Random forest was an optimization model based on bagging. The final result is merged and output by training multiple tree models. It used bootstrap aggregation and randomization of predictors to achieve high prediction accuracy and capture nonlinear dependencies [26, 27]. Extra Trees Regressor differs from traditional decision trees in the way they are constructed. When looking for the best split to split a sample of nodes into two groups, a random split is done for each of the randomly selected features (max_features), and the best split among them is chosen.

Adding Boost can further reveal secondary learning for prediction errors and improve the accuracy of prediction results. Ada Boost Regressor has the ability of adaptive enhancement. The samples that were wrongly classified by the previous primary classifier will be strengthened, and all the weighted samples will be used to train the next primary classifier again. At the same time, a new weak classifier is added in each round until a predetermined sufficiently small error rate is reached or a pre-specified maximum number of iterations is reached. However, the error of the Ada boost regressor model was quite high. This may be because the model required larger sample size and a smaller number of 291 training samples, which made it impossible to give full play to the advantages of the model. The larger the sample size was needed to improve the accuracy of the model. The production indicators of large-scale pig farms will not reach the maximum number, but there were corresponding production standards (see Additional file 2). When the depth of each regression tree was very small, the gradient boosting regressor model algorithm can export the same high accuracy as the deeper regression tree. Usually, the maximum depth of each regression tree was set to a smaller value to prevent overfitting [28]. The gradient promotion of regression tree produced a competitive, highly robust and interpretable process for regression and classification, which was especially suitable for mining dirty data. Gradient Boosting Regressor tree can improve the fitting effect by training multiple tree models of learning residuals. And as a nonlinear model, it can produce personalized calculation results. For Hist Gradient Boosting Regressor, discrete features can be converted into continuous features through histogram statistics, leading to the ability to directly process discrete features.

While these nine models are constantly evolving, that doesn't mean the latest model is the best. The optimal solution depends on many factors, such as the size of the dataset, the degree of dispersion, and the state of the data. In this study, due to the high correlation between each parameter (production factors and PSY), the nonlinear classification algorithm of the tree model was more suitable. Furthermore, given the upper bound of the data, the gradient boosting regressor model can reasonably deal with the ceiling effect (that was, the already high sensitivity will not be increased for the exposure above the average impact, just as the sensitivity will not be reduced for the exposure below the average effect). Therefore, the result with the smallest MAE, R^2^ and the narrowest 95% CI were obtained by the gradient boosting regressor model.

Our study concluded that WPL, MR7DW, PBAL, TPL and NPD were the most affecting production indicators of PSY, as previously reported [19]. Personalized analysis of 291 pig farms through the gradient boosting regulator model found that increasing the number of production days improved PSY in 1/3 of the pig farms. This may be because the longer production time, the more continuous and stable the production, and the more reasonable the parity structure of the sow herds. 92.1% of the investigated pig farms were newly built after the outbreak of African swine fever epidemic in 2018, and 70% were put into production after 2020. On one hand, it will be affected by the epidemic. On the other hand, the unstable personnel and insufficient quantity will also have a certain impact on the production performance. WPL and MR7DW had indirect effects on PSY because they directly affected and increased NPDs, thus reducing the number of piglets per sow per year [21] and PSY [29]. Sows in normal estrus after weaning were more likely to mate in 4–6 days after weaning in subsequent parity, and sows mated within 4–6 days after weaning had higher reproductive performance and longer lifetime productivity [30]. Therefore, the higher the MR7DW, the higher the lifetime reproductive performance of the PSY and even the sow.

## Conclusions

17 productive factors had a non-linear relationship with PSY. The main production factors related to PSY were WPL, MR7DW, PBAL, TBL and NPD. Compared with MAE, 95% CI and R^2^ among the nine algorithm models, the gradient boosting regressor was the optimal model to analyze the production factors that were non-linearly related to PSY, which can realize the personalized promotion of PSY for specific large-scale pig farms. Our practical approach has great application value prospects, looking for the optimal solution in every farm considering its own particular conditions, which lead to work individually in the most relevant factors in every case. 70% of 291 pig farms can improve PSY to various levels by increasing the production days, WPL and MR7DW.

## Supplementary Information

**Additional file 1: **The PSY improvement of each pig farm after the absolute value of 0.5 standard deviation of bottleneck calculated by gradient boosting regressor model.

**Additional file 2: **Production factor standard of 3000-scale pig farms.

## Data Availability

Due to producer confidentiality, the dataset and farm information are not publicly available.
