# Supplementary material for: Machine learning based personalized promotion strategy of piglets weaned per sow per year in large-scale pig farms
Source: Porcine Health Manag. 2022 Aug 10;8:37. doi: 10.1186/s40813-022-00280-z (PMC9364547; doi:10.1186/s40813-022-00280-z)
Supplement: Supplementary file 2 — Additional file 2: Production factor standard of 3000-scale pig farms. [file 40813_2022_280_MOESM2_ESM.docx]

**Additional file 2** Production factor standard of 3,000-scale pig farms

| No. | Production factors | Standard |
| --- | --- | --- |
| 1 | Actual stock | 2,700 |
| 2 | PSY | 25+ |
| 3 | Non-productive days | 35 |
| 4 | Mating sows / week | 146 |
| 5 | Farrowing litters | 120 |
| 6 | Conception rate | 90% |
| 7 | Farrowing rate | 85% |
| 8 | Number of piglets born alive per litter | 11.3 |
| 9 | Birth weight | 1.3-1.5 |
| 10 | Number of weaned piglets per litter | 10.2 |
| 11 | Survival rate at weaning | 90% |
| 12 | Weaning days | 24 |
| 13 | 21-day adjusted weight of piglets | 6.5-7 |
| 14 | Estrus rate after weaning | 85% |
| 15 | Slaughter rate | 90% |
| 16 | Feed conversion ratio | 267% |
